# The Expulsion of Tape-Worm by Pumpkin Seed

**Published:** 1855-05

**Authors:** 


### *The Expulsion of Tape-Worm by Pumpkin Seed.*
By John C. S. Monkur, M. D.

The following case is taken from my *Office Record of Medical and Surgical Cases,* 1855.

January 14, 1855, Mr. Grimes, aged 37 years, carpenter by occupation, is at the office to-day, with a vial containing *vermes cucurbitinus*—the tænia, or tape-worm. He states that since the 1st April, 1854, to the present time, January 14th, 1855, he has passed almost daily, from ten to thirty links of the worm in twenty-four hours. During this whole time, with the exception of the last two weeks previous to his present attendance at the office, he has enjoyed his usual good health; has had excellent appetite; not lost a day from his work: when his strength failed him, he became so weak as to be compelled to leave his daily labor, and with an entire loss of appetite, which obliged him to seek my advice to remove the worm. He is ordered the following: "Bruise six ounces of pumpkin seed thoroughly in a mortar, without removing the shells, and add water sufficiently to give by expression and straining, *one pint* of liquid. In the morning at 4 o'clock, he is to take half of the liquid, and follow it in two hours with two tablespoonsful of castor oil; he is at 1 o'clock, P. M., to take the other half, and at 3 o'clock, P. M., to take three tablespoonsful of the oil."

January 16. Mr. Grimes returns to-day, and has the worm in a bottle. He states that he took the medicines as directed, and at 4 o'clock yesterday evening they operated once; in fifteen minutes afterward the second time, which brought the entire worm, without other inconvenience than active purging from any other medicine. He passed first four joints; then followed an entire worm, which he measured by his carpenter's rule, and found it in length seventeen feet.

February 10, 1855. Mr. Grimes visits the office to-day, and reports that, from the expulsion of the worm to the present date, he has not seen or felt any of it, and is certain from its first appearance up to the time of his application for its removal, that links have passed from him night and day, asleep or awake, at work or play, from fifteen to twenty inches in twenty-four hours.

*Remarks.*—The case reported above is the second case the writer has had the opportunity to witness, and in both he was successful in the discharge of the worm by the administration of the pumpkin seed. The first case occurred in 1853, in which had been taken large, doses of spirits of turpentine, the male fern, and the new remedy, *kousso,* but ineffectually; when an *orgeat* of the seeds removed the entire worm, since which time the subject of it has had no return, and enjoyed a perfect state of health.

For the selection and employment of the pumpkin seed, the writer is indebted to the paper of Mr. Soule, of Boston, who has communicated his experience with it in volume xlv. of the Boston Medical and Surgical Journal. Other cases have since been reported in the same journal, of its successful action in the removal of tænia.

It may be remarked that care should be taken that the seed be fresh and good, (those grown in the West Indies are preferred,) and when properly prepared and duly administered, its purpose and efficiency may be relied on.

Baltimore, Md., Feb.—*N. J. Med. Reporter.*

